# Analysis of skewed X-chromosome inactivation in females with rheumatoid arthritis and autoimmune thyroid diseases

**DOI:** 10.1186/ar2759

**Published:** 2009-07-09

**Authors:** Ghazi Chabchoub, Elif Uz, Abdellatif Maalej, Chigdem A Mustafa, Ahmed Rebai, Mouna Mnif, Zouheir Bahloul, Nadir R Farid, Tayfun Ozcelik, Hammadi Ayadi

**Affiliations:** 1 Laboratoire de Génétique Moléculaire Humaine, Faculté de Médecine de Sfax, Avenue Majida Boulila, Sfax, 3029, Tunisie; 2 Department of Molecular Biology and Genetics, Faculty of Science. Bilkent University, Ankara, 06800, Turkey; 3 Unité de Bioinformatique, Centre de Biotechnologie de Sfax, Sfax, BP 3018, Tunisie; 4 Service d'Endocrinologie, Centre Hospitalo-universitaire Hédi Chaker de Sfax. Rue El-Ferdaous, Sfax, 3029, Tunisie; 5 Service de Médecine Interne, Centre Hospitalo-universitaire Hédi Chaker de Sfax. Rue El-Ferdaous, Sfax, 3029, Tunisie; 6 Osancor Biotech Inc, 31 Woodland Drive, Watford, Herts, WD17 3BY, UK; 7 Institute for Materials Science and Nanotechnology (UNAM), Bilkent University, Ankara, 06800, Turkey

## Abstract

**Introduction:**

The majority of autoimmune diseases such as rheumatoid arthritis (RA) and autoimmune thyroid diseases (AITDs) are characterized by a striking female predominance superimposed on a predisposing genetic background. The role of extremely skewed X-chromosome inactivation (XCI) has been questioned in the pathogenesis of several autoimmune diseases.

**Methods:**

We examined XCI profiles of females affected with RA (n = 106), AITDs (n = 145) and age-matched healthy women (n = 257). XCI analysis was performed by enzymatic digestion of DNA with a methylation sensitive enzyme (*Hpa*II) followed by PCR of a polymorphic CAG repeat in the androgen receptor (*AR*) gene. The XCI pattern was classified as skewed when 80% or more of the cells preferentially inactivated the same X-chromosome.

**Results:**

Skewed XCI was observed in 26 of the 76 informative RA patients (34.2%), 26 of the 100 informative AITDs patients (26%), and 19 of the 170 informative controls (11.2%) (*P *< 0.0001; *P *= 0.0015, respectively). More importantly, extremely skewed XCI, defined as > 90% inactivation of one allele, was present in 17 RA patients (22.4%), 14 AITDs patients (14.0%), and in only seven controls (4.1%, *P *< 0.0001; *P *= 0.0034, respectively). Stratifying RA patients according to laboratory profiles (rheumatoid factor and anti-citrullinated protein antibodies), clinical manifestations (erosive disease and nodules) and the presence of others autoimmune diseases did not reveal any statistical significance (*P *> 0.05).

**Conclusions:**

These results suggest a possible role for XCI mosaicism in the pathogenesis of RA and AITDs and may in part explain the female preponderance of these diseases.

## Introduction

It is postulated that the paternal and maternal antigens will be recognized by the immune system within the thymus, and T cells that have a high affinity for such antigens will be deleted by apoptosis [1-3]. The lack of exposure to a self-antigen in the thymus may lead to the presence of autoreactive T cells and increase the risk of autoimmunity [4]. In female mammalian cells, one of the two X-chromosomes is inactivated in early embryonic life [5]. Thus, females are mosaics for two cell populations, cells with either the paternal or the maternal X in the active form. X-chromosome choice is assumed to be random, and the result is generally 50% of cells expressing the paternal and the remaining 50% expressing the maternal genes [6]. A skewed X-chromosome inactivation (XCI) is a deviation from this ratio and is arbitrarily defined, for example, as a pattern where 80% or more of the cells inactivate the same X-chromosome [7]. This deviation may be the result of chance or genetic factors involved in the XCI or a selection process [8]. The existence of XCI in females offers a potential mechanism where by X-linked self-antigens may escape presentation in the thymus or in other peripheral sites that are involved in tolerance induction [9,10]. This has become an attractive candidate mechanism for breakdown of self-tolerance in autoimmune diseases. An association between skewed XCI and scleroderma was recently reported [11]. A higher frequency of a skewed XCI pattern was found in patients affected with autoimmune thyroid diseases (AITDs) compared with healthy controls, indicating that skewed XCI may be associated with a predisposing factor for the development of AITDs [12-14]. It was therefore of interest to study if there is an association between skewed XCI and rheumatoid arthritis (RA) as a non-organ-specific and AITDs as an organ-specific autoimmune disease. We investigated the peripheral blood XCI patterns of 106 females affected with RA, 145 females affected with AITDs and 257 controls in the Tunisian and Turkish populations. Extremely skewed XCI was found in the blood samples of female patients affected with RA and AITDs supporting the role of skewed XCI in female predisposition to autoimmune diseases.

## Materials and methods

### Patients and controls

#### RA sample

One hundred and six Tunisian women affected with RA were recruited into the study. All patients fulfilled the 1987 American College of Rheumatology criteria for RA [15]. A rheumatologist university fellow (ZB) reviewed all clinical data. The mean age was 47.6 ± 13.4 (mean ± standard deviation (SD)) years. The duration of the symptoms was 15 ± 8.9 years. The mean age of diagnostic was 40.3 ± 12 years. Among 106 RA patients, 65 were rheumatoid factor (RF) positive (61.3%), 70 were anti-citrullinated protein/peptide antibodies (ACPA) positive (66%), 15 presented with nodules (14.1%), and 70 presented with erosive disease (66%). Fifteen patients had another accompanying autoimmune diseases such as Sjögren's syndrome, type 1 diabetes, or autoimmune thyroid diseases.

#### AITDs sample

One hundred and forty-five Tunisian women affected with AITDs were included in the study. There were a total of 58 patients with Graves' disease (GD) and 87 patients with Hashimoto's thyroiditis (HT), which include 40 patients with the goitrous form. The mean age was 46.5 ± 14.5 years for AITDs patients (49.3 ± 13 years in HT patients and 44.6 ± 14 years in GD patients). The duration of the symptoms was 7.5 ± 4.6 years among the AITDs patients (6.8 ± 4.8 years in HT patients and 7.2 ± 4 years in GD patients). The mean age of diagnosis was 37.9 ± 15.1 years. The diagnosis of GD was based on the presence of biochemical hyperthyroidism as indicated by a decrease of thyroid-stimulating hormone (TSH), an increase of T4 levels, and positive TSH receptor antibody, in association with diffuse goiter or the presence of exophthalmos. The diagnosis of HT was based on the presence of thyroid hormone replaced primary hypothyroidism, defined as a TSH level above the upper limits associated with positive titers of thyroid autoantibodies (anti-thyroglobulin and/or anti-thyroid peroxidase) and with or without a palpable goiter.

#### Control group

Caucasian females, comprised of 97 Tunisian and 160 Turkish healthy unrelated volunteers, served as controls in our studies. The mean (± SD) age at analysis was 43.5 ± 15.3 years and 35 ± 9.9 years for Tunisian and Turkish controls, respectively. There was no clinical evidence or family history of autoimmune disease and inflammatory joint disease.

All individuals (patients and controls) provided informed consent. The ethics committee of the Centre Hospitalo-Universitaire Hédi Chaker de Sfax, Tunisie, and the Bilkent University, Ankara, Turkey approved the study protocol.

### Methods

#### Autoantibodies analysis

In AITDs patients, thyroid autoantibodies (anti-thyroglobulin and anti-thyroid peroxydase) were measured by ELISA and indirect immunofluorescence using commercially available kits (BINDAZYME™ Human EIA kits, Binding site Ltd, Birmingham, UK) with the respective normal ranges of 0 to 100 and 0 to 70 IU/mL.

The sera of RA patients obtained at the time of diagnosis were examined for RF by nephelometry and for ACPA by ELISA (second-generation test; Euro-Diagnostica, Arnhem, the Netherlands).

#### X-chromosome inactivation study

Genomic DNA was extracted from 10 ml of peripheral blood lymphocyte of patients and controls using standard methods [16]. Genotyping of a polymorphic site in the androgen receptor (*AR*) gene was performed and quantified to assess the XCI patterns as described [17]. The degree of skewing was estimated by an assay based on a methylation-sensitive *HpaII *restriction site located in exon 1 of the *AR *gene. This site is methylated on the inactive X, and unmethylated on the active X-chromosome. When the genomic DNA is cleaved with *HpaII *prior to PCR, only the methylated *AR *allele, which represents the inactive X-chromosome, is amplified. A polymorphic CAG repeat located within the amplified region is used to distinguish between the two alleles. For each patient and control two separate PCRs, with or without *Hpa*II treatment, were performed using the same set of primers. Densitometric analysis of the alleles was performed at least twice for each sample using the MultiAnalyst version 1.1 software (Bio-rad, Hercules, California, USA). A corrected ratio (CrR) was calculated by dividing the ratio of the predigested sample (upper/lower allele) by the ratio of the non-predigested sample for normalization of the ratios that were obtained from the densitometric analyses. The use of CrR compensates for preferential amplification of the shorter allele when the number of PCR cycles increases [18]. A skewed population is defined as a cell population with greater than 80% expression of one of the AR alleles. This corresponds to CrR values of less than 0.33 or more than three.

#### Statistical methods

The results from control and test groups in XCI studies were compared by chi-squared test with Yate's correction. Fisher's exact test was used when one cell had an expected count of less than one, or more than 20% of the cells had an expected count of less than five. *P *values of 0.05 or less were considerate to be significant. Significance of *P *value was assessed using a Bonferroni correction at 5% (a *P *value less 0.05/9 = 0.005) is considered significant.

## Results

XCI status was found to be informative in 76 of the 106 RA patients, 100 of the 145 AITDs patients and 170 of the 257 controls. Only those individuals whose alleles resolve adequately for densitometric analysis were included in the study. Skewed XCI (> 80% skewing) was observed in 26 of the 76 RA patients (34.2%), 26 of the 100 AITDs patients (26%), and 19 of the 170 controls (11.2%; *P *< 0.0001 and *P *= 0.0015). More importantly, the frequency of extremely skewed XCI (> 90% skewing) was 22.4% (17 of 76) in RA and 14.0% (14 of 100) in AITDs. These frequencies are both significantly higher than that of the control population, which is 4.1% (7 of 170; *P *< 0.0001 and *P *= 0.0034; Table 1). Subdividing AITDs patients according to clinical phenotype revealed that the frequency of skewed XCI was 35% (14 of 40, *P *= 0.0001) and 20% (12 of 60, *P *= 0.04) in GD and HT, respectively. Conversely, stratifying RA patients according to RF status, ACPA status, clinical manifestations (erosive disease and nodules) and others autoimmune diseases did not reveal a statistically significant difference (*P *> 0.05). Additionally, the comparison according to geographic origin showed a skewed XCI of RA patients compared with Tunisian controls (34.2% versus 19.5%; *P *= 0.03). However, difference was non-significant for AITDs subgroup (*P *> 0.05).

**Table 1:** Proportion of RA and AITDs patients and controls with skewed X-chromosome inactivation

	Number (%) observed with skewed		
	
Degree of skewing (%)	RA (n = 76)	AITDs (n = 100)	Control females (n = 170)
90+	17 (22.4)	14 (14)	7 (4.1)
80 to 89	9 (11.8)	12 (12)	12 (7.1)
70 to 79	11 (14.5)	23 (23)	29 (17.1)
60 to 69	28 (36.8)	22 (22)	36 (21.2)
50 to 59	11 (14.5)	29 (29)	86 (50.6)

For comparison by chi-squared *P *< 0.0001 and *P *= 0.0015 (> 80% skewing); *P *< 0.0001 and *P *= 0.0034 (90+% skewing) for patients with rheumatoid arthritis (RA) and autoimmune thyroid diseases (AITDs), respectively.

**Figure 1:**
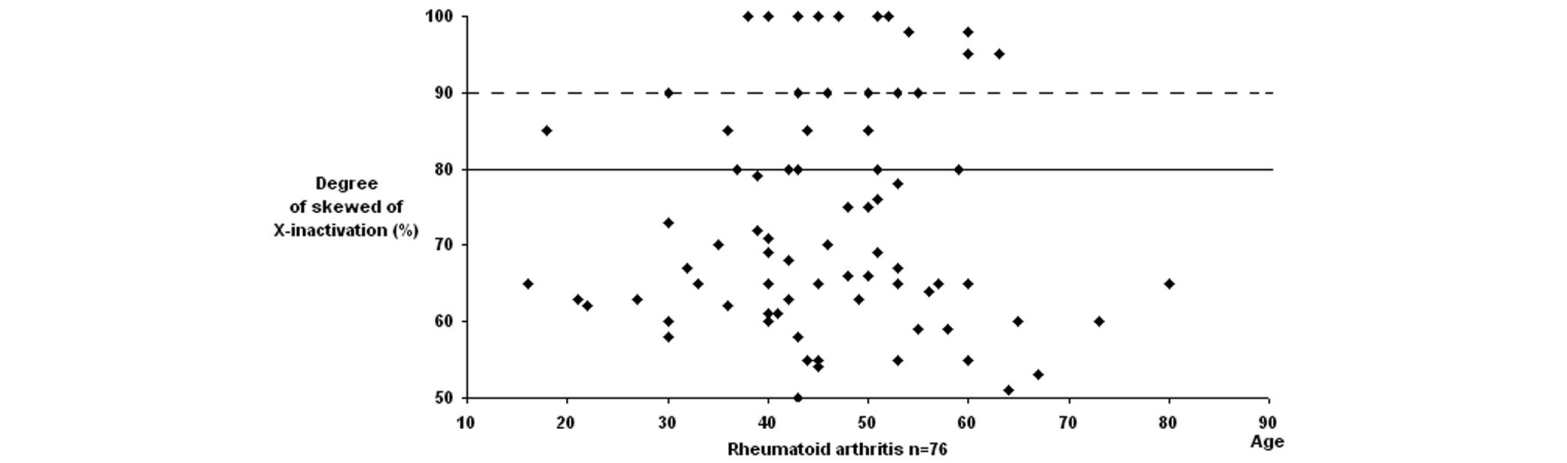
Distribution of X-chromosome inactivation patterns according to age in patients with rheumatoid arthritis.

**Figure 2:**
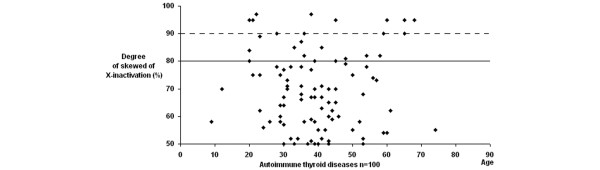
Distribution of X-chromosome inactivation patterns according to age in patients with autoimmune thyroid diseases.

**Figure 3:**
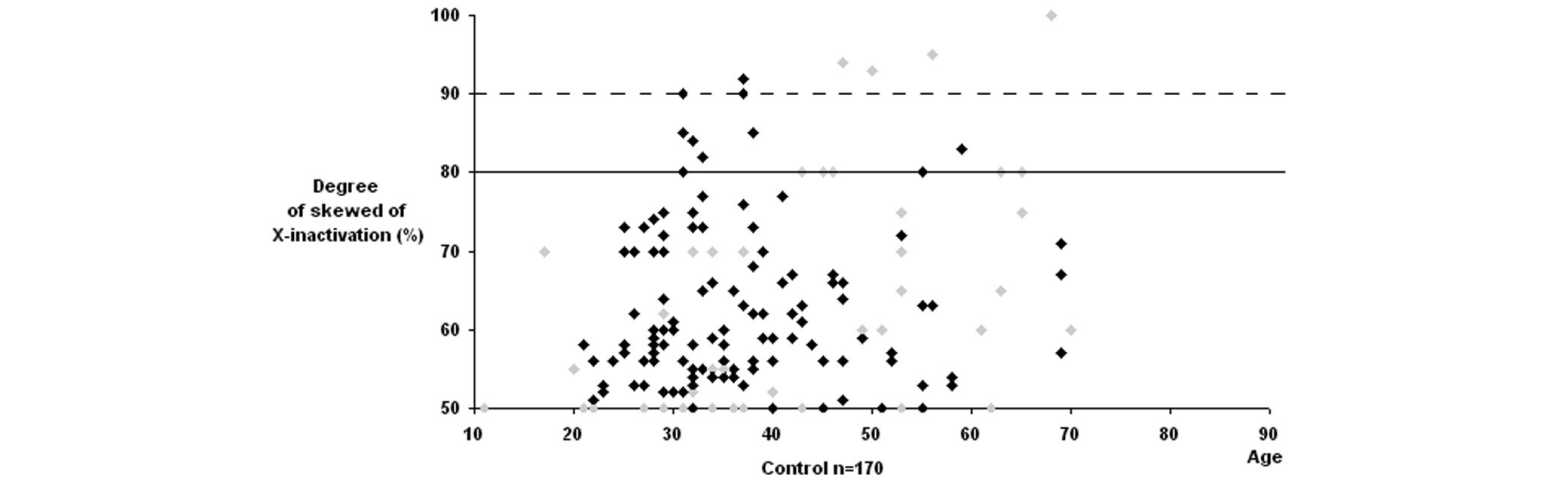
Distribution of X-chromosome inactivation patterns according to age control subjects. The control subjects were plotted according to geographic origin. Gray diamonds represent Tunisian controls and black diamonds represent Turkish controls.

Extremely skewed XCI have been reported in 1 to 2% of 20 to 40 year old women, and in 2 to 4% of 55 to 72 year old women [19]. The data for RA and AITDs patients is strikingly bimodal, we plotted the distribution of the X inactivation profiles according to age. However, we did not observe a shift toward the skewed range in older patients and controls (Figures 1, 2 and 3). Characteristics of the RA and AITDs patients with skewed XCI are shown in Tables 2 and 3.

**Table 2:** Characteristics of the patients with rheumatoid arthritis and skewed X-chromosome inactivation

Patient	Birth date	Disease onset	Pregnancy history	RF status	ACPA status	Other autoimmune disease	immunosuppressive therapy
90+% skewing							
1	1949	48	G7, P4, A3	+	+	GSG	MXT
2	1954	42	G5, P4, A1	+	+	-	Plaquenil
3	1945	52	G7, P7, A0	-	-	GSG	-
4	1946	40	G3, P2, A1	+	-	-	-
5	1956	30	G2, P2, A0	-	-	-	-
6	1946	40	G3, P2, A1	-	+	GSG	-
7	1945	40	G4, P4, A0	+	-	-	-
8	1945	39	G5, P5, A0	-	+	-	-
9	1941	49	G7, P5, A2	+	+	-	MXT
10	1947	49	G4, P3, A1	+	-	GSG	-
11	1945	58	G4, P2, A1	-	+	GSG	MXT
12	1950	40	G3, P2, A1	+	+	GSG	MXT
13	1943	53	G3, P3, A0	+	-	-	-
14	1961	35	G2, P1, A1	+	-	-	-
15	1937	38	G4, P4, A0	-	-	-	-
16	1941	45	G5, P3, A1	+	+	-	-
17	1947	43	G3, P2, A0	-	+	GSG	-
80 to 89% skewing							
18	1959	42	G5, P5, A0	+	+	-	MXT
19	1940	62	G0, P0, A0	-	-	-	-
20	1938	60	G9, P8, A1	+	+	GSG	MXT
21	1954	27	G0, P0, A0	+	+	GSG	-
22	1957	37	G5, P5, A0	+	+	GSG	-
23	1948	55	G9, P7, A0	+	-	-	MXT
24	1948	55	G0, P0, A0	-	-	-	-
25	1937	50	G3, P2, A1	-	+	GSG	-
26	1985	14	G0, P0, A0	+	-	-	-

A = spontaneous abortions; ACPA = anti-citrullinated protein/peptide antibodies; G = number of pregnancies; GSG = Sjögren's syndrome; MTX = methotrexate; P = para (pregnancies carried to term and delivered); RF = rheumatoid factor.

**Table 3:** Characteristics of the patients with autoimmune thyroid diseases and skewed X-chromosome inactivation

Patient	Birth date	Disease onset	Pregnancy history	Diagnostic	Auto antibodies
90+% skewing					
1	1978	22	G1, P1, A0	HT	+
2	1933	65	G11, P5, A0	HT	+
3	1969	20	G1, P1, A1	HT	+
4	1938	60	G3, P3, A0	GD	+
5	1943	45	G2, P2, A0	GD	+
6	1972	21	G2, P2, A0	HT	+
7	1964	36	G2, P2, A0	HT	+
8	1924	65	G9, P9, A0	HT	+
9	1940	59	G10, P10, A0	HT	+
10	1969	28	G4, P4, A0	GD	+
11	1979	20	G0, P0, A0	GD	-
12	1931	68	G13, P13, A0	HT	-
13	1943	59	G1, P1, A0	GD	-
14	1946	42	G2, P2, A0	GD	-
80 to 89% skewing					
15	1969	23	G2, P2, A0	HT	+
16	1950	41	G3, P2, A1	GD	+
17	1980	20	G0, P0, A0	HT	+
18	1945	54	G4, P3, A0	HT	+
19	1962	36	G7, P2, A5	HT	+
20	1954	48	G3, P3, A0	GD	+
21	1984	20	G0, P0, A0	GD	+
22	1952	45	G2, P2, A0	GD	-
23	1941	58	G5, P5, A0	HT	-
24	1953	39	G2, P2, A0	GD	-
25	1947	48	G1, P1, A0	HT	-
26	1969	43	G2, P1, A1	GD	-

A = spontaneous abortions; G = number of pregnancies; GD = Graves' disease; HT = Hashimoto's thyroiditis; P = para (pregnancies carried to term and delivered).

At the time of sample collection, 66 patients affected with RA were being treated with immunosuppressive therapies (methotrexate 10 to 15 mg once a week, n = 33; D-penicillamine 300 mg/day, n = 17; plaquenil 400 to 600 mg/day, n = 16). Among 76 informative patients, 46 were received immunosuppressive agents (61%). A major concern with the observed XCI patterns among RA patients was that concomitant immunosuppressive therapy could influence the results, as has been observed in feline hematopoietic cells [20]. Analysis of the data on XCI patterns according to immunosuppressive therapy did not reveal a statistically significant association between RA patients treated with methotrexate and controls (*P *= 0.52).

## Discussion

The majority of human autoimmune diseases are characterized by female predominance. RA and AITDs have a female:male ratio of approximately 3:1 and 9:1, respectively [21]. Sex hormone influences have been suggested to explain this phenomenon because the X-chromosome contains a considerable number of sex and immune-related genes such as *AR*, IL2 receptor gamma chain, *CD40 *ligand and *FOXP3 *[22,23]. These genes are essential in determining sex hormone levels and, more importantly, immune tolerance [24]. The contribution of genetics to sex differences in autoimmune diseases is currently unexplored. An alternative explanation for the female predominance has been recently proposed with the finding of an enhanced skewed XCI in peripheral bloods cells of female patients with autoimmune diseases [11-14]. The present study tests the hypothesis that skewed XCI would be more prevalent in females affected with autoimmune diseases than in female control individuals. Therefore, we simultaneously examined skewed XCI in 106 patients affected with RA and 145 patients affected with AITDs. The control group consisted of 170 female age-matched healthy individuals. We have demonstrated a significantly higher prevalence of extremely skewed XCI in blood cell of females affected with RA and AITDs compared with the control group (*P *< 0.0001; *P *= 0.0015, respectively), indicating a possible role of XCI in the etiology of autoimmune diseases, and in the female preponderance of RA and AITDs.

Skewed XCI was more commonly expected in peripheral blood mononuclear cells due to the very high rate of turnover of blood cells compared with other solid tissues [25]. Then, we have examined XCI in peripheral blood mononuclear cells of patients affected with RA and AITDs, and we found a higher incidence of skewed XCI in those patients. We also tested the relationship between XCI and AITDs phenotypes (GD and HT). A skewed XCI was associated with both GD and HT (*P *= 0.0001 and *P *= 0.04). Although, our results suggest the involvement of XCI in female predisposition to RA and AITDs, this hypothesis still to be confirmed in specific tissue, because our analysis was performed in DNA from blood, and this may not be a representative tissue for all autoimmune diseases [26,27] and there may exist locally skewed XCI in the thymus. Moreover, this study can be complicated by existing differences in peripheral blood mononuclear cells constituents in RA versus healthy controls. The XCI distribution in both Tunisian and Turkish controls (Figure 3) according to age showed that 19.5% (9 of 46) have a skewed XCI in Tunisian controls which have a mean age of 43.5 years, whereas only 8% (10 of 124) in Turkish controls with a younger mean age (35 years). This result suggests the importance of age in the difference of XCI skewing.

Our results are in agreement with those reported by Ozçelik and colleagues on 110 unrelated Turkish female AITDs patients and 160 female controls that showed a greater proportion of a skewed pattern of XCI (34%) than in controls (8%; *P *< 0.0001) [13]. Indeed, supporting data have been reported by Brix and colleagues, which assessed that the prevalence of skewed XCI in female twins affected with AITDs was 34% but only 11% in controls (*P *= 0.003) and by Yin and colleagues (*P *= 0.004) [12-14]. Similar positive result was described in other autoimmune diseases such as scleroderma [11]. In addition, our results are the first report that describes a significant association between extremely skewed XCI and RA. Conversely, examination of XCI pattern of 58 Caucasian female patients affected with multiple sclerosis, 46 with systemic lupus erythematosus, 18 with juvenile RA and 45 with type 1 diabetes mellitus and 30 healthy women did not reveal skewed XCI patterns [28]. Despite extensive efforts of XCI analysis in different autoimmune diseases and populations, this hypothesis remains to be confirmed because there is no apparent autoimmunity directed against protein antigens encoded on the X chromosome and the fact that, for many autoimmune diseases, we found a female predominance in inbred mice models having two identical X chromosomes and therefore no 'foreign' antigens from the XCI [29].

In humans, it was reported that XCI process was genetically controlled by genes located on X chromosome [30]. It has also been suggested that genes on the X chromosome might show linkage with AITD and RA [31,32]. Thus, the observed association between skewed XCI and AITD and RA is not causal but could be explained by linkage disequilibrium between mutation responsible for XCI process and AITD and RA susceptibility polymorphisms. In addition, numerous environmental risk factors such as tobacco smoking, hormones, diet, drugs, toxins and/or infections are important in determining whether an individual will develop autoimmune diseases [33]. In fact, environmental agents are able to amplify autoimmunity in genetically susceptible individuals and to break tolerance in genetically resistant individuals, there by increasing the risk of developing autoimmune diseases [34]. The interaction between genetic and environmental factors remains to be achieved in order to evaluate the involvement of each component in the development of such autoimmune reactions.

## Conclusions

We suggest a possible role of XCI mosaicism in the pathogenesis of RA and AITDs. However, the process of XCI needs to be considered as a potential factor in the predominance of females in most autoimmune diseases. It would also be of interest first to study the XCI pattern in females affected with other autoimmune diseases and second to test the XCI patterns of many cell types.

## Abbreviations

ACPA: anti-citrullinated protein/peptide antibodies; AITDs: autoimmune thyroid diseases; AR: androgen receptor; CrR: corrected ratio; ELISA: enzyme-linked immunosorbent assay; GD: Graves' disease; HT: Hashimoto's thyroiditis; IL: interleukin; PCR: polymerase chain reaction; RA: rheumatoid arthritis; RF: Rheumatoid factor; SD: standard deviation; TSH: thyroid stimulating hormone; XCI: X-chromosome inactivation.

## Competing interests

The authors declare that they have no competing interests.

## Authors' contributions

GC carried out the molecular genetic study, performed the statistical analysis and wrote the manuscript. EU participated in the experimental work and the statistical analysis. AM participated in the design of the study and helped to draft the manuscript. AR participated in the statistical analysis. MM made pathological diagnosis and performed clinical data analyses. CAM participated in the molecular genetic study. ZB made pathological diagnosis, conducted sampling procedures, and performed clinical and rheumatological data analyses. TO conceived of the study, and participated in its design and coordination and helped to draft the manuscript. HA participated in the coordination of the study and revised the manuscript. All authors read and approved the final manuscript.
